# Paeoniflorin Ameliorates Colonic Fibrosis in Rats with Postinfectious Irritable Bowel Syndrome by Inhibiting the Leptin/LepRb Pathway

**DOI:** 10.1155/2022/6010858

**Published:** 2022-10-03

**Authors:** Ya-Qing Tian, Sheng-Peng Zhang, Kun-Li Zhang, Di Cao, Yi-Jun Zheng, Ping Liu, Hui-Hui Zhou, Ya-Ning Wu, Qi-Xiang Xu, Xiao-Ping Liu, Xu-Dong Tang, Yong-Qiu Zheng, Feng-Yun Wang

**Affiliations:** ^1^Provincial Engineering Laboratory for Screening and Re-Evaluation of Active Compounds of Herbal Medicines in Southern Anhui, Teaching and Research Section of Traditional Chinese Medicine, School of Pharmacy, Wannan Medical College, Wuhu 241000, Anhui, China; ^2^China Academy of Chinese Medical Sciences, Beijing, China

## Abstract

Postinfectious irritable bowel syndrome (PI-IBS) is a highly prevalent gastrointestinal disorder associated with immune dysregulation and depression- and anxiety-like behaviors. Through traditional medicine, the active ingredient of Paeoniae Radix called paeoniflorin (PF) was previously found to prevent the symptoms of PI-IBS. However, there is limited information on the effects of PF on intestinal function and depression- and anxiety-like symptoms in PI-IBS animal models. Here, we aimed to determine the effects of PF treatment on the symptoms of PI-IBS in a rat model. The PI-IBS rat model was established via early postnatal sibling deprivation (EPSD), trinitrobenzenesulfonic acid (TNBS), and chronic unpredictable mild stress (CUMS) stimulation and then treated with different dosages of PF (10, 20, and 40 mg/kg) and leptin (1 and 10 mg/kg). The fecal water content and body weight were measured to evaluate the intestinal function, while the two-bottle test for sucrose intake, open field test (OFT), and elevated plus maze test (EMT) were performed to assess behavioral changes. The serum leptin levels were also measured using an enzyme-linked immunosorbent assay. Furthermore, the expressions of leptin and its receptor, LepRb, were detected in colonic mucosal tissues through an immunohistochemical assay. The activation of the PI3K/AKT signaling pathway and the expression of brain-derived neurotrophic factor (BDNF) were also detected via western blotting. After the experimental period, the PI-IBS rats presented decreased body weight and increased fecal water content, which coincided with elevated leptin levels and heightened depression- and anxiety-like behaviors (e.g., low sucrose intake, less frequency in the center areas during OFT, and fewer activities in the open arms during EMT). However, the PF treatment ameliorated these observed symptoms. Furthermore, PF not only inhibited leptin/LepRb expression but also reduced the PI3K/AKT phosphorylation and BDNF expression in PI-IBS rats. Notably, cotreatment with leptin (10 mg/kg) reduced the effects of PF (20 mg/kg) on colonic fibrosis, leptin/LepRb expression, and PI3K/AKT activation. Therefore, our findings suggest that leptin is targeted by PF via the leptin/LepRb pathway, consequently ameliorating the symptoms of PI-IBS. Our study also contributes novel insights for elucidating the pharmacological action of PF on gastrointestinal disorders and may be used for the clinical treatment of PI-IBS in the future.

## 1. Introduction

In this modern age, the living habits and diets of humans are greatly influenced by the fast-paced and high-stress environment, contributing to chronic psychosocial disorders with high-risk factors for gastrointestinal diseases [1]. For example, postinfectious irritable bowel syndrome (PI-IBS) is described as a prevalent multifactorial disease with acute infectious enteritis accompanied by recurrent abdominal pain, altered bowel habits, fever, bellyache, vomiting, diarrhea, gut flora, immune dysregulation, and depression- and anxiety-like disorders [2–4]. Currently, to manage these symptoms, different methods have been attempted, including probiotic intervention, moxibustion, psychotherapy, exercise, and antidepressant medication [5]. Although there are several drugs available for treating PI-IBS, effective and approved treatments for one or more of the symptoms of PI-IBS are still needed.

Previous studies reported that leptin might play an important role in the immune-mediated bowel dysfunction in IBS patients [6, 7]. Leptin is a 16 kDa nonglycosylated peptide hormone that exists in the rat intestine from the duodenum to the colon. The presence of a leptin receptor (LepRb) in the small intestine suggests that luminal leptin potentially modulates the immune system and gastrointestinal function [8, 9]. There is also evidence that leptin and LepRb participate in the pathogenesis of depression and anxiety [10, 11]. Additionally, previous studies reported that dextran sulfate sodium-induced trinitrobenzenesulfonic acid (TNBS) colitis is attenuated in mice with leptin deficiency [12, 13]. Further investigation confirmed that the plasma levels of leptin are significantly increased in the chronic unpredictable mild stress (CUMS) rat model [14]. Similarly, high serum levels of leptin are observed in patients with major depression [15]. Notably, infusing leptin into the murine ventral tegmental area could activate the leptin/LepRb downstream pathway and result in reduced anxiogenic behaviors [16]. Additionally, IBS patients display altered cytokine profiles, including the elevated circulating concentrations of the proinflammatory cytokine interleukin-6, which bears similarities in structural homology and intracellular signaling to leptin [6].

In fact, the therapeutic effects of medicines against PI-IBS are very hard to evaluate because of lacking preclinical animal models. TNBS was prepared by high-pressure sterilization, and the success rate of PI-IBS model establishment was more than 95% [17, 18]. However, PI-IBS is a common gastrointestinal disorder and is considered to be a functional disease because there appears to be no associated anatomical defect. Stress and psychological factors are thought to have an important role in IBS [19]. We develop a rat model of PI-IBS, not only with associated symptoms of immune-mediated bowel dysfunction of PI-IBS, such as recurrent abdominal pain, diarrhea, and gut flora but also with the pathogenesis of depression-and anxiety-like disorders. The model was established through complex stimulation with early postnatal sibling deprivation (EPSD), TNBS, and CUMS [20]. However, the exact role of leptin in a rat model with a gastrointestinal disorder, specifically induced via complex stimulation of EPSD, TNBS, and chronic unpredictable mild stress (CUMS), is unknown.

In traditional Chinese medicine, the roots of peony (*Paeonia lactiflora* Pall) are used for preparing various herbal formulas to treat depression, including “Danggui-Shaoyao-San” and “Xiaoyao powder” [21, 22]. Its main active component, called paeoniflorin (PF), a water-soluble monoterpene glycoside, was discovered to exert remarkable anti-inflammatory, immunopharmacological, antidepressant, and antifibrotic effects in multiple animal models [23–25]. PF also has neuroprotective effects related to depression in animal models, such as CUMS [26, 27]. In addition, PF (30 mg/kg and 60 mg/kg) potentially exerts a neuroprotective effect against CUMS-induced hippocampal damage in rats by regulating the extracellular regulated protein kinase (ERK)-cAMP-response element binding protein (CREB) signaling pathway [24]. Furthermore, the herbal prescription Chang'an II, which also contains PF (paeoniflorin 38.35 mg/g), reportedly attenuates the symptoms of IBS at dosages of 2.85, 5.71, and 11.42 g/kg [28].

In the present study, we aimed to determine the effects of PF on intestinal function and depression- and anxiety-like behaviors in a PI-IBS animal model. Additionally, the effects of PF on the leptin/LepRb pathway were investigated in vivo.

## 2. Materials and Methods

### 2.1. Animals

Adult pregnant Sprague-Dawley rats (190–210 g) were purchased from Zhejiang Weitong Lihua Laboratory Animal Technology Co., Ltd. (animal license number SCXK; Zhejiang; 2019–0001). All rats were handled in accordance with the regulations of the National Institutes of Health, and the study was approved by the Institutional Animal Care and Use Committee of Wannan Medical College. Throughout the acclimatization and treatment periods, all animals had access to food and water *ad libitum* and were maintained on a 12 h light/dark cycle at 21 ± 2°C and 45 ± 10% relative humidity, except during the CUMS experiment and two-bottle test. The rats were also housed under specific pathogen-free conditions.

### 2.2. Establishment of the PI-IBS Rat Model

**Figure 1:**
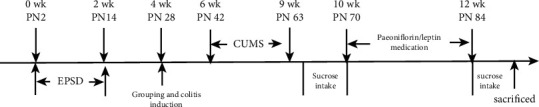
Experimental protocol.

Since PI-IBS is an inflammatory immune disease, only newborn male rats were used. Based on previously published methods [28, 29], the rat model for PI-IBS was established using a multistimulation paradigm composed of EPSD, TNBS, and CUMS. Briefly, the litters were moved from the maternity cages to the adjacent cages at 9:00–12:00 AM during postnatal day 2 (PN2) to PN14. After pentobarbital anesthesia, colitis was induced in PN28 rats through intrarectal administration of 0.8 mL TNBS solution (20 mg per rat) in 50% ethanol, following a previously described method [30]. In contrast, the control rats were administered with 0.8 mL 50% ethanol as a vehicle. All solutions were delivered via a soft catheter and introduced 8 cm above the anus. After recovery from TNBS treatment for 2 weeks, the following procedures were performed: (1) water-fasting for 24 h; (2) fasting for 24 h; (3) reverse day/night cycle (dark from 7:00 to 19:00 and light from 19:00 to 7:00 the next day); (4) induction of cold stress for 5 min (the rats were placed in a transparent barrel containing ice water at 4°C at a depth of 15 cm); (5) induction of heat stress for 5 min (the rats were placed in a thermostat at 45°C); (6) pain induction (the rats were placed in an observation cage and their tails were clipped 1 cm from the distal tip, with appropriate strength to make the rat scream); (7) horizontal oscillation for 15 min (the rats were placed in a high-speed horizontal oscillator [110 r/min]). Each procedure was performed daily for 21 consecutive days (Figure 1) [31, 32].

### 2.3. Drug Treatment

Based on the previous study [24, 28], the dose of PF that we used in our PI-IBS models included the range of dosage (10, 20, and 40 mg/kg). We also allotted a 14-day pretreatment period to ensure sufficient time for the onset of PF activity. The PF (C23H28O11; MW: 480.45; purity: ≥95% [HPLC]; LD50: 9,530 mg/kg) obtained from Ningbo Dekang Biologic Product Co., Ltd. (Ningbo, China) was dissolved in distilled water and used for daily intragastric administration for 10–12 weeks. Recombinant rat leptin (R&D Systems, Minneapolis, MN, USA) was dissolved in sterile saline at a concentration of 1 mg/mL and intraperitoneally injected into the animals daily (at a dose of 1 and 10 mg/kg body weight) for 10–12 weeks. To determine the effective dosage of PF, the rats were randomly divided into five groups: (1) Normal, (2) PI-IBS, (3) PF (10 mg/kg) + PI-IBS, (4) PF (20 mg/kg) + PI-IBS, and (5) PF (40 mg/kg) + PI-IBS. To assess the effect of leptin on the mechanism of PF, the rats were randomly divided again into five groups: (1) Normal, (2) PI-IBS, (3) PF (20 mg/kg) + PI-IBS, (4) PF (20 mg/kg) + PI-IBS + leptin (1 mg/kg), and (5) PF (20 mg/kg) + PI-IBS + leptin (10 mg/kg).

### 2.4. Open Field Test (OFT)

The rats were individually placed in the middle of an open field apparatus (height: 40.0 cm; length: 100.0 cm; width: 100.0 cm) between 8:00 and 11:00 AM. As described in a previous study [33], 25 squares (20.0 cm × 20.0 cm) were drawn on the floor, and the number of times that the rats crossed between squares was counted by two observers (blind to the experimental groups) over a 10 min period. Crossing squares, as a measure of locomotion, was counted when the rat had moved all four legs from one quadrant to another. After each trial, the open field apparatus was carefully cleaned.

### 2.5. Two-Bottle Test

To evaluate the effect of PF on depression, a two-bottle test for sucrose intake was performed as described in a previous study [34]. The rats were separately housed and supplied for 24 h with one bottle filled with tap water and another with 1% sucrose solution. To balance side preference, the locations of the two bottles were switched after 12 h. Following 24 h of fasting and water abstinence, the experimental process was immediately initiated after training completion. The rats were given 1 h of access to the two bottles, and the consumption in each bottle was recorded. The percentage of sucrose consumption was calculated as (sucrose intake/total intake) × 100%.

### 2.6. Elevated Plus Maze Test (EMT)

Rats were placed at the intersection of a maze, consisting of two open and two closed arms, and left for 5 minutes. The 4 arms meet in a 5 cm × 5 cm central square open area which enabled the rats to freely enter each arm of the maze. Time spent in the open arms, number of times entering the open arms, and distance traveled in each arm by the rats were analyzed to evaluate anxiety‐like behavior.

### 2.7. Histological Examination

One week after the two-bottle test, the rat colons were removed and fixed with paraformaldehyde. The colons were subsequently embedded in paraffin and cut into 5 *μ*m-thick sections. For hematoxylin and eosin (H&E) and Masson trichrome staining, the slices were developed using 3,3′-diaminobenzidine (DAB) and then counterstained either with hematoxylin or using Trichrome, Gomori One-Step, Fast Green Stain Kit (Newcomer Supply, Middleton, WI, USA).

### 2.8. Immunohistochemical (IHC) Assay

The colon samples were fixed with 4% formalin and embedded in paraffin. Tissue slices (5 *μ*m thick) were prepared for IHC staining. The leptin/LepRb antibody (1:200; Thermofisher Scientific, Waltham, MA, USA) was used as the primary antibody. With Image J software, we calculated the integrated optical density (IOD) of each section from 8 different 400x magnified fields. For IHC assessment, the entire tissue section was scanned and scored by two independent pathologists.

### 2.9. Measurement of Serum Leptin Levels

Venous blood samples (1 mL) were collected, centrifuged at 2,000 rpm for 10 min, and then stored at −80°C for temporary storage. The levels of leptin in the serum were measured using enzyme-linked immunosorbent assay (ELISA) kits, following the manufacturers' protocols (Abcam, Cambridge, UK). The sample and standard dilutions were made using the experimental media.

### 2.10. Western Blot Analysis

Proteins were extracted from tissue samples or cell lysates using RIPA buffer (150 mM NaCl, 50 mM Tris-Cl, 1 mM EGTA, 1% [v/v] Triton X-100, 0.1% [w/v] SDS, and 1% [w/v] sodium deoxycholate; pH 8.0). To evaluate the effects of PF on leptin/LepRb downstream signaling, the PI-IBS rats were sacrificed, and colon homogenates were prepared. Protein concentrations were determined using a protein assay solution (Bio-Rad Laboratories, Hercules, CA, USA). Equivalent amounts of proteins were denatured in protein loading buffer, resolved on 10% sodium dodecyl sulfate-polyacrylamide gel electrophoresis (SDS-PAGE) gels, and subsequently transferred to polyvinylidene difluoride (PVDF) membranes (Millipore, Billerica, MA, USA) via electroblotting. The PVDF membranes were blocked with 5% nonfat milk in Tris-buffered saline/Tween buffer for 1 h and incubated overnight at 4°C with the antibodies against phosphatidylinositol 3-kinase (PI3K, 1 : 1,000; Cell Signaling Technology, Danvers, MA, USA), p-PI3K (1 : 1,000; Cell Signaling Technology), protein kinase B (AKT, 1 : 1,000; Cell Signaling Technology), p-AKT (1 : 1,000; Cell Signaling Technology), brain-derived neurotrophic factor (BDNF, 1 : 1,000; Cell Signaling Technology), and glyceraldehyde 3-phosphate dehydrogenase (GAPDH, 1 : 1,000; Cell Signaling Technology). The signals were detected using Pierce™ Electrochemiluminescence (ECL) Detection Reagent (Thermofisher Scientific), following the manufacturer's protocol.

### 2.11. Statistical Analysis

The data obtained are presented as means ± SD. A significant difference was determined using a one-way analysis of variance (ANOVA), followed by Tukey's test for multiple comparisons. A nonparametric test was used to compare the band density values between groups. Statistical significance was set at *P* < 0.05.

## 3. Results

### 3.1. Effects of PF Treatment on the Body Weight, Fecal Water Content, and Depression-and Anxiety-like Behaviors of PI-IBS Rats

**Figure 2:**
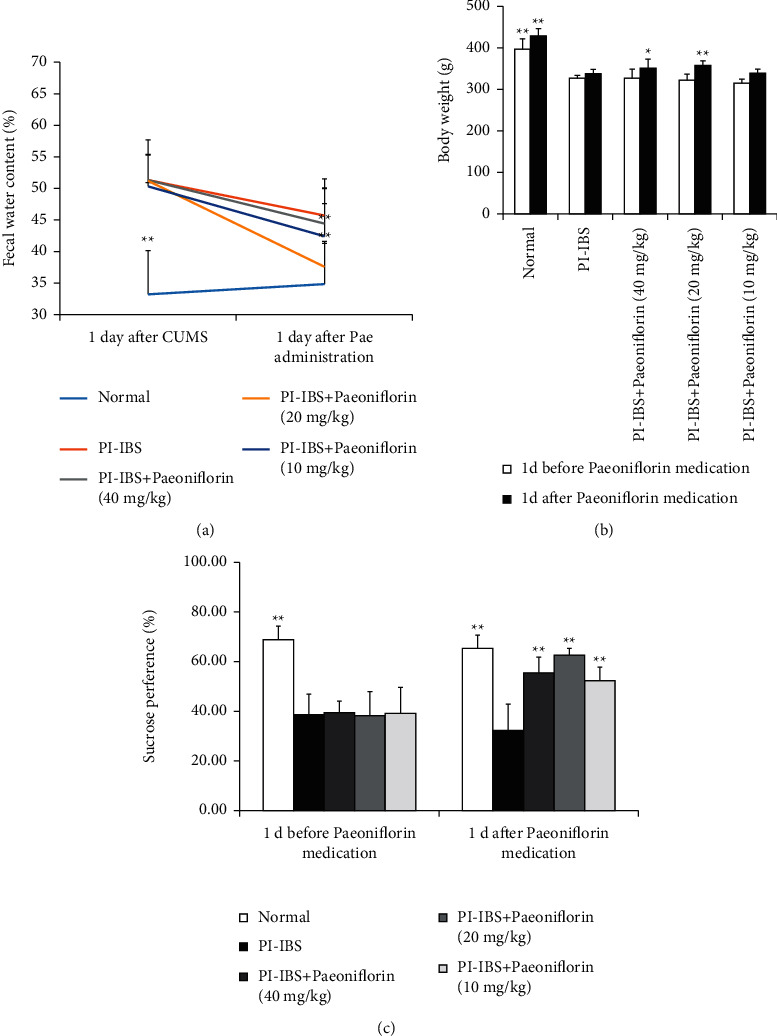
PF treatment ameliorates the symptoms in PI-IBS rats. (a) Reduced fecal water content and suppressed body weight gain are observed in the experimental groups one day after CUMS and PF treatment. (b) No difference is observed between experimental groups one day before and after PF treatment. (c) Reduced sucrose consumption is observed between experimental groups one day before and after PF treatment. Data are presented as means ± SEM (*n* = 6 per group);  ^*∗*^*P* < 0.05 and  ^*∗∗*^*P* < 0.01 versus PI-IBS group.

**Figure 3:**
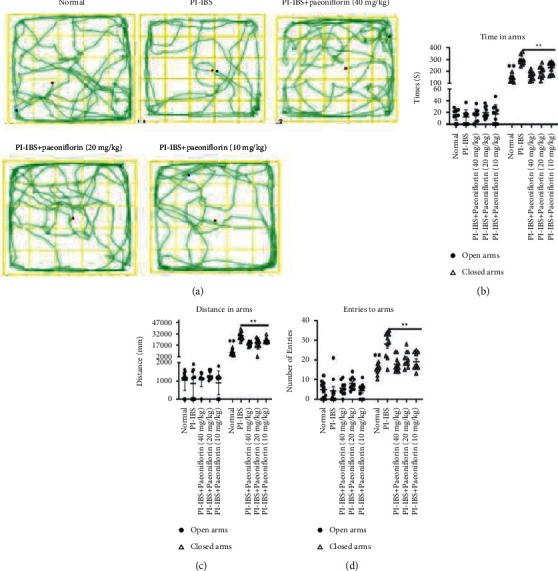
Behavioral assessment (OFT and EMT) of PI-IBS rats treated with different doses of PF. (a) There is no significant difference in the total distance between the experimental groups during an open field test. Time spent in open or closed arms (b), distance traveled in open or closed arms (c), and the number of entries to open or closed arms (d) in an elevated plus maze. Data were expressed as mean ± SD.  ^*∗∗*^*P* < 0.01 versus the PI-IBS group.

To determine the effect of PF treatment in PI-IBS rats, the body weight and fecal pellets were first measured to assess the successful induction of the PI-IBS model. In addition, the two-bottle test, OFT and EMT were performed to observe the depression-and anxiety-like behaviors. After the EPSD, TNBS, and CUMS experiments, the rats exhibited lower body weight and higher fecal water content compared to the control rats, indicating the successful establishment of the PI-IBS model. Notably, compared to the PI-IBS group, the two PF + PI-IBS groups (20 and 40 mg/kg) showed a considerable increase in body weight and decreased fecal water content (Figures 2(a) and 2(b)). These results suggest that the symptoms in PI-IBS rats were alleviated after PF treatment. The results of the two-bottle test also determined the sucrose consumption in PI-IBS rats (Figure 2(c)). The ANOVA analysis revealed a significant decrease in sucrose preference after the EPSD, TNBS, and CUMS experiments. However, the preference for sucrose increased in PI-IBS rats after PF treatment, suggesting the PF-induced amelioration of PI-IBS symptoms (e.g., reduced intestinal function, depression). The ANOVA analysis also revealed the significant negative effects of the EPSD, TNBS, and CUMS experiments on the behavior of PI-IBS rats during OFT (Figure 3(a) and Table 1). However, the frequency of staying in the center areas increased in the PF + PI-IBS groups. Furthermore, there were no differences between the total distances crossed by the model and PF-treated groups. In the EMT, PF + PI-IBS rats and PI-IBS rats spent a similar amount of time in the open arms. The PI-IBS rats tended to spend more time, move more distance, and have more entries into the closed arms compared to the PF-treated rats (Figures 3(b)–3(d)). The results imply that the complex stimulation with early postnatal sibling deprivation (EPSD), TNBS, and CUMS caused the highest level of anxiety which was reduced by PF treatment.

### 3.2. Effects of PF Treatment on Colonic Inflammation and Fibrosis in PI-IBS Rats

**Figure 4:**
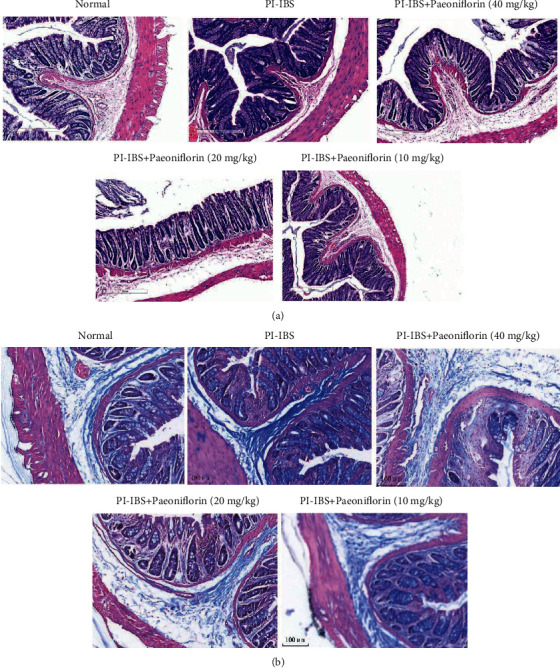
(a) Histological examination through H&E staining shows that PF treatment relieves the inflammatory infiltration in the colon of PI-IBS rats. Scale bar = 300 *μ*m. (b) Masson's trichrome staining demonstrates that PF treatment reduces fibrosis in the colon of PI-IBS rats. Scale bar = 100 *μ*m. PF treatment Prevents Leptin/LepRb-mediated pathway activation in PI-IBS rats.

The results of the histological examination showed that the active inflammation and chronic mucosal injury in PI-IBS rats were significantly more serious compared to those of the control. Further analysis using Masson's trichrome staining revealed the excessive fibrotic remodeling in the submucosa and smooth muscle of PI-IBS rats. After PF treatment, examination of the colons from medium- (20 mg/kg) and high-dose (40 mg/kg) PF-treated rats revealed their normal appearance, but with less collagen in the submucosa and smooth muscle (Figures 4(a) and 4(b)), suggesting that the occurrence of fibroplasia was inhibited by PF. Overall, these results indicate that the protective effects of PF against PI-IBS injury produce long-term improvement in the intestinal structure and function in this immunoinflammatory rat model.

**Figure 5:**
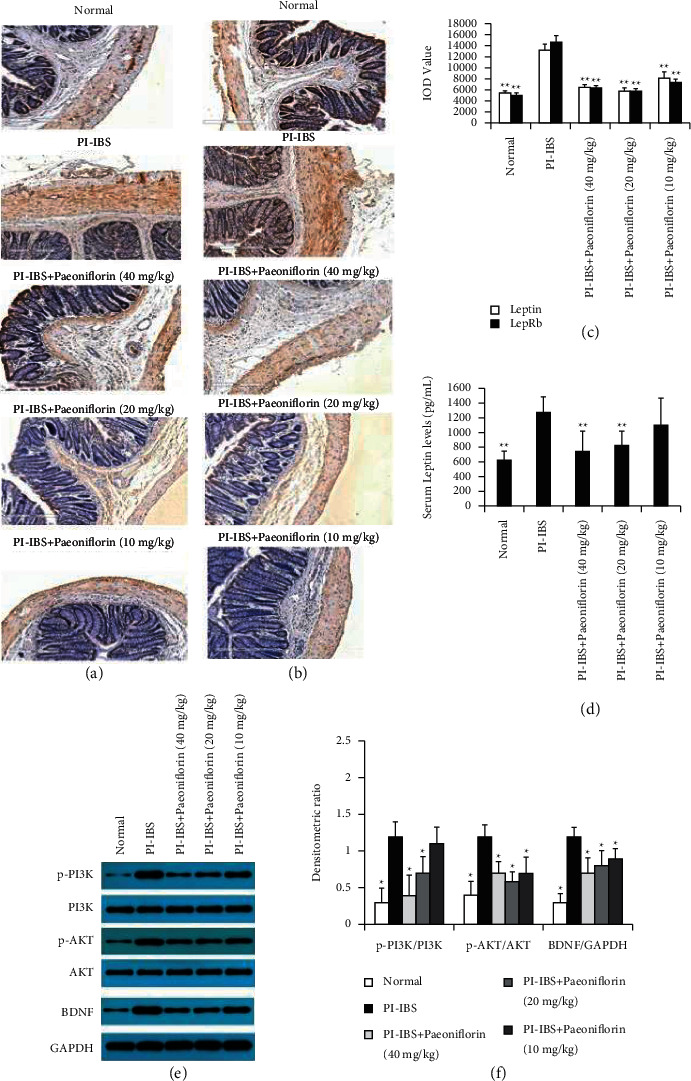
PF treatment inhibits the leptin/LepRb pathway activation in PI-IBS rats. ((a)-(b)) Evaluation of leptin (a) and LepRb (b) expression in the colon tissues via IHC staining. Scale bar = 300 *μ*m. (c) IOD value of leptin and LepRb. (d) Analysis of the leptin serum levels using an ELISA kit shows the reduced leptin serum levels after PF administration (20 and 40 mg/kg). (e)-(f) Western blot analysis of p-PI3K/PI3K, p-AKT/AKT, and BDNF/GAPDH protein expression. (e) Representative gel images of p-PI3K, PI3K, p-AKT, AKT, and BDNF, with GAPDH as control. (f) The bar graph shows the quantitative evaluation of p-PI3K/PI3K, p-AKT/AKT, and BDNF/GAPDH expression (*n* = 3). ^*∗*^*P* < 0.05 and ^*∗∗*^*P* < 0.01 versus PI-IBS group.

Since the PF treatment significantly inhibited the PI-IBS-induced inflammation and fibrosis, we further investigated leptin and LepRb, which can effectively inhibit PI-IBS in vivo, using IHC assay and ELISA to detect their expression levels in the rat intestine and serum. The IHC assay results demonstrated that leptin and LepRb expression levels were significantly increased after stimulation via EPSD, TNBS, and CUMS, which corroborated the high leptin content in the serum. Compared to PI-IBS rats, oral administration of PF (20 and 40 mg/kg) resulted in reduced leptin and LepRb expression in the colon and serum of PF + PI-IBS rats (Figures 5(a)–5(d)). Consequently, the activation of the leptin/LepRb downstream pathway, specifically the PI3K-AKT pathway, was significantly suppressed in the PF + PI-IBS groups. Furthermore, the PI-IBS-induced expression of BDNF was markedly reduced in the PF-treated groups compared to that in the PI-IBS model group (Figures 5(e) and 5(f)). These results strongly suggest that the regulation of leptin and LepRb expression, PI3K-AKT pathway activation, and BDNF expression are essential to the PF-induced recovery process in PI-IBS rats.

### 3.3. Administration of Leptin Reduces the Protective Effects of PF Treatment in PI-IBS Rats

**Figure 6:**
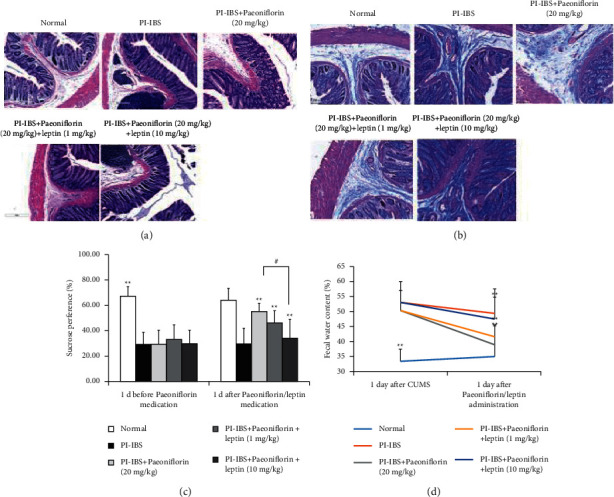
Leptin treatment reduces the anti-inflammatory and antifibrotic effects of PF on PI-IBS rats. (a) Representative images of H&E-stained colon tissues show the degree of inflammation. Scale bar = 300 *μ*m. (b) Masson's trichrome-stained colon tissues show the degree of fibrosis. Scale bar = 100 *μ*m. (c) Sucrose consumption between experimental groups one day before and after PF treatment. (d) Fecal water content between experimental groups one day before and after PF + leptin treatment.  ^*∗∗*^*P* < 0.01 versus PI-IBS group; ^*∗*^*P* < 0.05 and ^##^*P* < 0.01 versus PI-IBS + PF (20 mg/kg) group.

**Figure 7:**
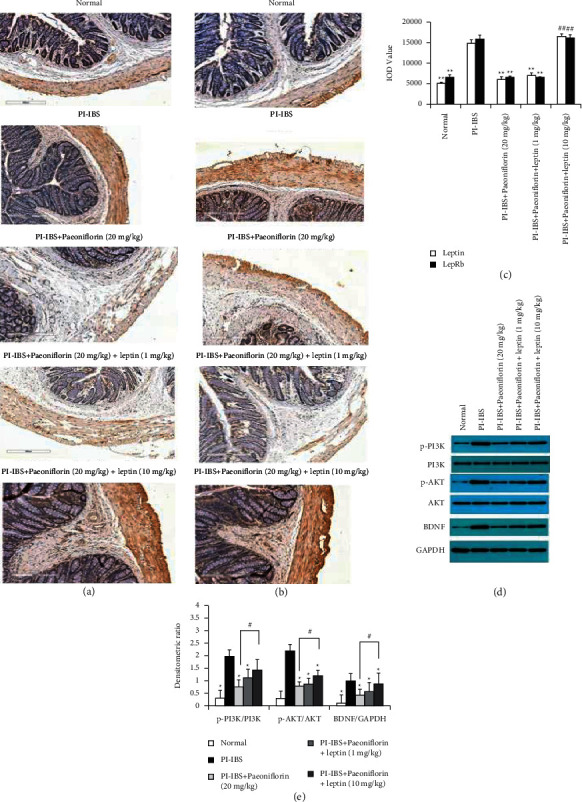
Leptin administration attenuates the effects of PF by inhibiting the activation of leptin/LepRb downstream signaling. ((a)-(b)) Evaluation of leptin (a) and LepRb (b) expression in the colon tissues via IHC staining. Scale bar = 300 *μ*m. (c) IOD value of leptin and LepRb. ((d)-(e)) Western blot analysis of p-PI3K/PI3K, p-AKT/AKT, and BDNF/GAPDH protein expression. (d) Representative gel images of p-PI3K, PI3K, p-AKT, AKT, and BDNF, with GAPDH as control. (e) Bar graph showing the quantitative evaluation of p-PI3K/PI3K, p-AKT/AKT, and BDNF/GAPDH expression (*n* = 3). ^*∗*^*P* < 0.05 and  ^*∗∗*^*P* < 0.01 versus PI-IBS group; ^#^*P* < 0.05 versus PI-IBS + PF (20 mg/kg) group.

To further explore the mechanism of PF via the leptin/LepRb pathway, additional experiments were performed to determine whether leptin and LepRb can be used as possible molecular targets for PF. The results showed that the cotreatment of leptin and PF could sufficiently inhibit the protective effects of PF in the PF + PI-IBS + leptin groups. In specific, colonic inflammation and fibrosis were aggravated to almost the same levels as that of model rats (nontreated) and coincided with increased fecal water content and decreased sucrose preference (Figure 6). Furthermore, compared to PI-IBS rats treated with PF only, the PI-IBS rats treated with 10 mg/kg leptin and 20 mg/kg PF displayed higher leptin and LepRb expression levels, subsequently inducing the activation of the PI3K-AKT pathway. In contrast, the PI-IBS rats treated with 1 mg/kg leptin and 20 mg/kg PF exhibited features that were similar to those treated with PF only (Figure 7). These results imply that the leptin/LepRb signaling pathway is the direct inhibitory target of PF, resulting in the amelioration of PI-IBS symptoms, including excessive fibrotic remodeling in the submucosa and smooth muscle, chronic mucosal injury, reduced intestinal function, and depression- and anxiety-like behaviors.

## 4. Discussion

As the main active ingredient in peony [35], the pharmacological mechanisms of PF have been extensively studied in various inflammatory diseases, such as psoriasis, Parkinson's disease, and rheumatoid arthritis [36–38]. In the present study, two novel findings were obtained: (1) the expression levels of leptin and LepRb were significantly elevated in the colon after EPSD, TNBS, and CUMS stimulation, and (2) the protective effects of PF in PI-IBS rats were related to the inhibition of the leptin/LepRb pathway.

As an important regulator during the pathogenesis of depression and inflammation, the circulating levels of leptin were found to be positively correlated with the severity of depression [39]. Additionally, Leptin can reportedly either promote or suppress inflammation in the intestines [40, 41]. Furthermore, elevated leptin and LepRb levels enhance fibrogenesis in the heart, liver, lungs, and other organs [42–44]. In the present study, we discovered that the expression levels of leptin and LepRb were significantly increased in the colon after EPSD, TNBS, and CUMS stimulation, which was consistent with the high leptin content in the serum, suggesting that leptin influences the development of colonic inflammation, fibrosis, and depression-like behavior in PI-IBS rats.

The PI-IBS model established through complex stimulation with EPSD, TNBS, and CUMS exhibits clinical symptoms that most closely match those of PI-IBS patients, such as interstitial cystitis, visceral hypersensitivity, and psychiatric disturbances, which were assessed in the present study. We then observed that PF treatment could improve the low body weight and high fecal water content in PI-IBS rats. Simultaneously, the PF treatment enhanced the preference for sucrose during the two-bottle test and the frequency in the center areas during the OFT and reduced the times, distance, and frequency of rats entering the closed arms during the EMT, indicating that PF ameliorated the depression- and anxiety-like behaviors in PI-IBS rats. The results of the histological examination confirmed that PF exerted anti-inflammatory and antifibrotic effects on PI-IBS rats. Moreover, the PF treatment was found to alleviate the inflammation, fibrosis, and depression-like behavior in PI-IBS rats by downregulating the leptin and LepRb expression in the colon and serum. Notably, further investigation revealed that the protective effects of PF on PI-IBS rats were fully reversed after cotreatment with 10 mg/kg leptin. Thus, our findings demonstrate that the protective effects of PF on PI-IBS rats are directly related to the inhibition of the leptin/LepRb pathway.

Previous studies reported that the binding of leptin to LepRb resulted in the suppression of the glycogen synthase kinase 3*β* (GSK3*β*)/*β*-catenin signaling, which was characterized by the increased phosphorylation of PI3K and AKT and expression of BDNF [45–47]. Hence, we also attempted to determine the role of the leptin-mediated PI3K/AKT pathway in the PI-IBS rat model. The PI3K/AKT pathway is essential for the maintenance of intestinal function and for regulating a variety of important biological processes [48, 49]. As a key molecule, PI3K was also found to participate in intestinal inflammation and fibrosis [50, 51]. The activation of PI3K is known to enhance the phosphorylation of AKT and to negatively regulate the activity of GSK3*β* [52]. Similarly, we observed that the administration of PF inhibited the activation of the leptin/LepRb-mediated PI3K/AKT pathway in PI-IBS rats, which was associated with reduced depression- and anxiety-like behaviors, suggesting that PF ameliorates the symptoms in PI-IBS rats by suppressing the leptin/LepRb downstream pathway.

In addition, BDNF is reported to exist in the intestinal mucosa and enteric nervous system, which are modulated by the PI3K/AKT/GSK3*β* pathway. Interestingly, BDNF is a neuromodulator associated with the clinical features of IBS, such as abdominal pain and discomfort [53–57]. Furthermore, TNBS-induced colon inflammation is associated with high BDNF expression and the stimulation of sensory neurons in the dorsal root ganglia [58]. In the present study, the oral administration of PF inhibited the BDNF expression in the colon of PI-IBS rats. However, treatment with leptin (10 mg/kg) reversed the effects of PF on BDNF expression, demonstrating that PF ameliorates the PI-IBS symptoms by suppressing the BDNF expression in the colon.

Thus, the leptin/LepRb pathway may be associated with the multifactorial effects of PF against gastrointestinal disorders and depression- and anxiety-like behaviors in PI-IBS rats. However, further research is required to fully elucidate the association between PF, leptin, and PI-IBS. In summary, leptin has been confirmed as the target of PF in ameliorating the symptoms in PI-IBS rats, such as excessive fibrotic remodeling in the submucosa and depression- and anxiety-like behaviors via the inhibited activation of the leptin/LepRb downstream pathway—the PI3K/AKT pathway. Taken together, our findings implicate leptin/LepRb as an important target of PF and identify it as a potential biomarker for human patients with gastrointestinal disorders.

## 5. Conclusion

Therefore, we hypothesize that PF ameliorates the symptoms in PI-IBS rats by suppressing the leptin/LepRb downstream pathway.

## Figures and Tables

**Table 1:** Effects of PF on the behavior of rats in the open field test (*n* = 6).

Group	Dose (mg.kg/d)	Total distance (cm)	Distance in the center (cm)
Normal	—	21825.18 ± 2398.25	1145.4 ± 292.27^*∗∗*^
PI-IBS	—	17456.94 ± 11374.55	316.72 ± 134.92
PI-IBS + paeoniflorin	40	22618.44 ± 3154.50	645.16 ± 106.96^*∗∗*^
PI-IBS + paeoniflorin	20	18921.52 ± 2230.14	640.95 ± 25.29^*∗∗*^
PI-IBS + paeoniflorin	10	19698.85 ± 5617.20	771.52 ± 109.06^*∗∗*^

Data were expressed as mean ± SD.  ^*∗∗*^*P* < 0.01 versus the PI-IBS group.

## Data Availability

No data were used in this study.
